# Growth differentiation factor-11 downregulates steroidogenic acute regulatory protein expression through ALK5-mediated SMAD3 signaling pathway in human granulosa-lutein cells

**DOI:** 10.1186/s12958-022-00912-7

**Published:** 2022-02-19

**Authors:** Qiongqiong Jia, Boqun Liu, Xuan Dang, Yanjie Guo, Xiaoyu Han, Tinglin Song, Jung-Chien Cheng, Lanlan Fang

**Affiliations:** Center for Reproductive Medicine, Henan Key Laboratory of Reproduction and Genetics, The First Affiliated Hospital of Zhengzhou University, 40, Daxue Road, Zhengzhou, 450052 China

**Keywords:** GDF-11, SMAD, StAR, Granulosa cells, PCOS

## Abstract

**Background:**

Growth differentiation factor-11 (GDF-11) belongs to the transforming growth factor-β (TGF-β) superfamily. To date, the expression of GDF-11 in the ovary and its role in regulating ovarian function are completely unknown. Ovarian granulosa cell-mediated steroidogenesis plays a pivotal role in maintaining normal female reproductive function. GDF-11 and GDF-8 share high sequence similarity and exhibit many similar features and functions. Steroidogenic acute regulatory protein (StAR) regulates the rate-limiting step in steroidogenesis and its expression can be downregulated by GDF-8. Polycystic ovary syndrome (PCOS) is the most common cause of female infertility. The expression levels of GDF-8 are upregulated in the human follicular fluid and granulosa-lutein (hGL) cells of PCOS patients. However, whether similar results can be observed for the GDF-11 needs to be determined.

**Methods:**

The effect of GDF-11 on StAR expression and the underlying molecular mechanisms were explored by a series of in vitro experiments in a primary culture of hGL cells obtained from patients undergoing in vitro fertilization (IVF) treatment. Human follicular fluid samples were obtained from 36 non-PCOS patients and 36 PCOS patients. GDF-11 levels in follicular fluid were measured by ELISA.

**Results:**

GDF-11 downregulates StAR expression, whereas the expression levels of the P450 side-chain cleavage enzyme (P450scc) and 3β-hydroxysteroid dehydrogenase (3β-HSD) are not affected by GDF-11 in hGL cells. Using pharmacological inhibitors and a siRNA-mediated approach, we reveal that ALK5 but not ALK4 mediates the suppressive effect of GDF-11 on StAR expression. Although GDF-11 activates both SMAD2 and SMAD3 signaling pathways, only SMAD3 is involved in the GDF-11-induced downregulation of StAR expression. In addition, we show that SMAD1/5/8, ERK1/2, and PI3K/AKT signaling pathways are not activated by GDF-11 in hGL cells. RT-qPCR and ELISA detect GDF-11 mRNA expression in hGL cells and GDF-11 protein expression in human follicular fluid, respectively. Interestingly, unlike GDF-8, the expression levels of GDF-11 are not varied in hGL cells and follicular fluid between non-PCOS and PCOS patients.

**Conclusions:**

This study increases the understanding of the biological function of GDF-11 and provides important insights into the regulation of ovarian steroidogenesis.

## Background

Steroidogenesis is a complex process that involves several subcellular compartments, multiple enzymes, substrates, and products whereby cholesterol is converted to biologically active steroid hormones. The human ovary is a steroidogenic organ that produces estradiol (E2) and progesterone (P4) under the regulation of gonadotropins during the menstrual cycle. E2 and P4 play pivotal roles in the regulation of sex differentiation and reproductive function. The key regulatory step involved in ovarian steroidogenesis is the regulation of the transport of cholesterol from the outer to the inner membrane of the mitochondria, where the cytochrome P450 side-chain cleavage enzyme (P450scc), the enzyme catalyzes the first step of steroidogenesis, is located [1]. It has been well characterized that cholesterol transfer from the outer to the inner membrane of the mitochondria is mediated by the steroidogenic acute regulatory protein (StAR) [1, 2]. Ovarian StAR expression is regulated by gonadotropins through the cAMP-PKA signaling pathway [3].

Growth differentiation factor-11 (GDF-11), also known as bone morphogenetic protein-11 (BMP-11), belongs to the transforming growth factor-β (TGF-β) superfamily and is expressed during embryogenesis [4–6]. Deletion of the *Gdf11* gene in mice results in perinatal lethality and shows that *Gdf11* is important in anterior/posterior axial patterning during embryonic development [7]. In humans, the expression of GDF-11 can be detected in nearly all major organs and tissues with the highest levels of that in the spleen, kidney, and brain [8, 9]. Now it is known that GDF-11 regulates the development of various organs and plays a role in age-related physiological and pathological functions. In addition, aberrant circulating levels of GDF-11 have been reported to be associated with the risk of cardiovascular diseases and cancers [10]. GDF-11 and GDF-8 share 89% sequence identity within their mature and signaling domain and have redundant functions in regulating skeletal patterning in mice but not in the regulation of skeletal muscle size [5, 11]. It has been shown that GDF-11 and GDF-8 signals through three type I receptors, activin receptor-like kinase 4 (ALK4), ALK5, and ALK7, and two activin type II receptors, ActRIIA and ActRIIB. Generally, upon binding to their receptors, GDF-11 and GDF-8 activate canonical SMAD2/3 and non-canonical ERK1/2, p38, and JNK signaling pathways [12, 13].

Despite their high similarity in protein sequence, utilization of membrane receptors, and activated intracellular signaling pathways, increasing evidence has suggested that GDF-11 and GDF-8 may have distinct functions in a context-dependent manner [12, 13]. GDF-8 and its receptors are expressed in the human ovary and can suppress the expression of StAR through ALK5-mediated SMAD3 and ERK1/2 signaling pathways in human granulosa-lutein (hGL) cells [14–16]. Polycystic ovary syndrome (PCOS), a hormonal disorder, is the most common cause of infertility in women [17]. We and another group have demonstrated that expression levels of GDF-8 in follicular fluid and hGL cells of PCOS patients are significantly higher than those of non-PCOS patients [16, 18, 19]. In addition, high GDF-8 levels in human follicular fluid are associated with a low pregnancy rate in the in vitro fertilization (IVF) patients with PCOS [19]. To date, the role of GDF-11 in female reproductive function is completely unknown. Given the remarkable similarities in biological properties between GDF-8 and GDF-11, the present study was designed to explore the effect and related underlying molecular mechanisms of GDF-11 on StAR expression in hGL cells and to examine whether the expression levels of GDF-11 in follicular fluid and hGL cells are varied between non-PCOS and PCOS patients.

## Methods

### Antibodies and reagents

The StAR (#sc-166821) and α-tubulin (#sc-23948) antibodies were obtained from Santa Cruz Biotechnology. The ALK5 (#PA5-78198) antibody was obtained from Invitrogen. The phospho-SMAD1/5/8 (#13820), SMAD1 (#6944), phospho-SMAD2 (#3108), phospho-SMAD3 (#9520), SMAD2 (#3103), SMAD3 (#9523), SMAD4 (#38,454), phospho-ERK1/2 (#9106), ERK1/2 (#9102), phospho-AKT (#9271), and AKT (#9272) antibodies were obtained from Cell Signaling Technology. The recombinant human GDF-11, GDF-8, BMP-4, and amphiregulin were obtained from R&D Systems. SB431542, a potent ALK4, ALK5, and ALK7 inhibitor [20], was obtained from Sigma. Dorsomorphin and DMH-1 were obtained from Cayman.

### Serum and follicular fluid samples collections and hormones measurements in non-PCOS and PCOS patients

The study received institutional approval and was carried out in accordance with the guidelines from the Zhengzhou University Research Ethics Board. Written informed consent was obtained from all of the subjects before participation in the study. Human serum and follicular fluid samples were obtained from 36 non-PCOS patients and 36 PCOS patients during IVF treatment. The diagnosis of PCOS was based on the revised Rotterdam diagnostic criteria for PCOS [21]. None of the women had been prescribed any medications before enrollment. Causes of infertility were tubal obstruction or male infertility. Patients with endometriosis, diminished ovarian reserve, chromosome abnormality, or hydrosalpinx were excluded from the study. All patients were treated with a standard long protocol. At the mid-luteal phase, the gonadotropin-releasing hormone (GnRH) agonist triptorelin (0.1 mg) (Ipsen Pharma Biotech), was administered subcutaneously daily. Approximately 14 days after GnRH agonist injection was started, recombinant FSH (Gonal-F; Merck) was administered daily at a dosage of 150–300 IU. When at least three follicles had reached 18 mm, hCG (10,000 IU, Livzon) was injected. Oocyte retrieval was scheduled approximately 34–36 h after hCG injection by transvaginal ultrasound-guided follicular aspiration. The follicular fluid was collected when the oocytes were retrieved. Only the first follicular fluid aspirate without blood or flushing solution was used for analysis. After 10 min of centrifugation at 1200 rpm, the supernatant was stored at -80 °C until further analysis. GDF-11 protein levels in follicular fluid were measured using an ELISA Kit (#E-EL-H1908, Elabscience) as per the manufacturer’s instructions. The analytical sensitivity of GDF-11 ELISA was 9.38 pg/mL. Both intra-CV and inter-CV were < 10%. Basal levels of follicle-stimulating hormone (FSH), luteinizing hormone (LH), estradiol (E2), progesterone (P4), and testosterone (T) in serum were measured on day 2 or 3 of the menstrual cycle from each subject by the electrochemiluminescence immunoassay (Roche). The characteristics of patients including age, body mass index (BMI), and antral follicle count (AFC) were obtained from the electronic medical records.

### Primary human granulosa-lutein (hGL) cell culture and treatments

The primary hGL cells were purified by density centrifugation from follicular aspirates collected from patients undergoing oocyte retrieval as previously described [22, 23]. Cells were cultured in a humidified atmosphere containing 5% CO_2_ and 95% air at 37 °C in phenol red-free Dulbecco’s Modified Eagle Medium/nutrient mixture F-12 Ham medium (DMEM/F-12; Gibco) supplemented with 10% charcoal/dextran-treated FBS (HyClone), 100 U/mL of penicillin, and 100 μg/mL of streptomycin sulfate (Boster). Primary hGL cells were serum-starved in a medium containing 0.5% charcoal/dextran-treated FBS for 24 h to induce quiescence before treatments. All treatments were performed in a medium containing 0.5% charcoal/dextran-treated FBS. The recombinant human GDF-11 was solubilized in phosphate-buffered saline (PBS). SB431542, dorsomorphin, and DMH-1 were dissolved in dimethyl sulfoxide (DMSO). All groups in each experiment were exposed to all the relevant vehicles for that experiment. Individual primary cultures were composed of cells from one individual patient. Each experiment was repeated at least three times and each time used cells derived from different patients.

### Reverse transcription quantitative real-time PCR (RT-qPCR)

Total RNA was extracted with the RNeasy Plus Mini Kit (QIAGEN) according to the manufacturer’s instructions. The concentration of RNA was measured by the NanoDrop Spectrophotometers. RNA (1 μg) was reverse-transcribed into first-strand cDNA with the iScript Reverse Transcription Kit (Bio-Rad Laboratories). Each 20 μL qPCR reaction contained 1X SYBR Green PCR Master Mix (Applied Biosystems, Shanghai, China), 60 ng of cDNA, and 250 nM of each specific primer. The primers used were 5′-AAA CTT ACG TGG CTA CTC AGC ATC-3′ (sense) and 5′-GAC CTG GTT GAT GAT GCT CTT G-3′ (antisense) for steroidogenic acute regulatory protein (StAR); 5′-CAG GAG GGG TGG ACA CGA C-3′ (sense) and 5′-AGG TTG CGT GCC ATC TCA TAC-3′ (antisense) for P450 side-chain cleavage enzyme (P450scc); 5′-GCC TTC CAG ACC AGA ATT GAG AGA-3′ (sense) and 5′-TCC TTC AAG TAC AGT CAG CTT GGT-3′ (antisense) for 3β-hydroxysteroid dehydrogenase (3β-HSD); 5'-TCT CTC CAC CTC AGG GTC TG-3' (sense) and 5'-GCC ATA CTT CCC CAA ACC GA-3' (antisense) for ALK4; 5'-GTT AAG GCC AAA TAT CCC AAA CA-3' (sense) and 5'-ATA ATT TTA GCC ATT ACT CTC AAG G-3' (antisense) for ALK5; 5'-CCG AAA TGC CAC GGT AGA AA-3' (sense) and 5'-GGG CTC TGC ACA AAG ATT GC-3' (antisense) for SMAD2; 5'-CCC CAG CAC ATA ATA ACT TGG-3' (sense) and 5'-AGG AGA TGG AGC ACC AGA AG-3' (antisense) for SMAD3; 5'-TCC ACA GGA CAG AAG CCA TT-3' (sense) and 5'-GTC ACT AAG GCA CCT GAC CC-3' (antisense) for SMAD4; 5'-CTG GAG GAG GAC GAG TAC CA-3' (sense) and 5'-GAA CAT CAC CTT GGG GCT GA-3' (antisense) for GDF-11; and 5′-GAG TCA ACG GAT TTG GTC GT-3′ (sense) and 5'-GAC AAG CTT CCC GTT CTC AG-3' (antisense) for GAPDH. RT-qPCR was performed using an Applied Biosystems QuantStudio 12 K Flex Real-Time PCR system equipped with a 96-well optical reaction plate. The specificity of each assay was validated by melting curve analysis and by agarose gel electrophoresis of the PCR products. All of the RT-qPCR experiments were run in triplicate, and a mean value was used to determine the mRNA levels. Water and mRNA without RT were used as negative controls. Relative quantification of the mRNA levels was performed using the comparative Ct method with GAPDH as the reference gene and using the formula 2^–∆∆Ct^.

### Western blot

Cells were lysed in cell lysis buffer (Cell Signaling Technology) supplemented with a protease inhibitor cocktail (Sigma). The protein concentration was analyzed by the BCA protein assay kit (Pierce, Thermo Scientific). Equal amounts (50 µg) of protein were separated by SDS polyacrylamide gel electrophoresis and transferred onto PVDF membranes. After 1 h blocking with 5% non-fat dry milk in Tris-buffered saline (TBS), the membranes were incubated overnight at 4 °C with primary antibodies diluted in 5% non-fat milk/TBS. Following primary antibody incubation, the membranes were incubated with appropriate HRP-conjugated secondary antibodies. Immunoreactive bands were detected using an enhanced chemiluminescent substrate (Bio-Rad Laboratories) and imaged with a ChemiDoc MP Imager (Bio-Rad Laboratories). Band intensities were quantified using the Scion Image software.

### Small interfering RNA (siRNA) transfection

To knockdown endogenous ALK4, ALK5, SMAD2, SMAD3, and SMAD4, cells were transfected with 100 nM ON-TARGETplus SMARTpool siRNA targeting specific genes (Dharmacon) using Lipofectamine RNAiMAX (Invitrogen). The ON-TARGETplus siCONTROL NON-TARGETING pool siRNA (Dharmacon) was used as the transfection control.

### Statistical analysis

The results are presented as the mean ± SEM or mean ± SD of at least three independent experiments. All statistical analyses were analyzed by PRISM software. Multiple comparisons were analyzed using one-way ANOVA followed by Tukey’s multiple comparison test. For experiments involving only two groups, the results were analyzed by *t* test. A significant difference was defined as *p* < 0.05.

## Results

### GDF-11 downregulates StAR expression in hGL cells

**Fig. 1:**
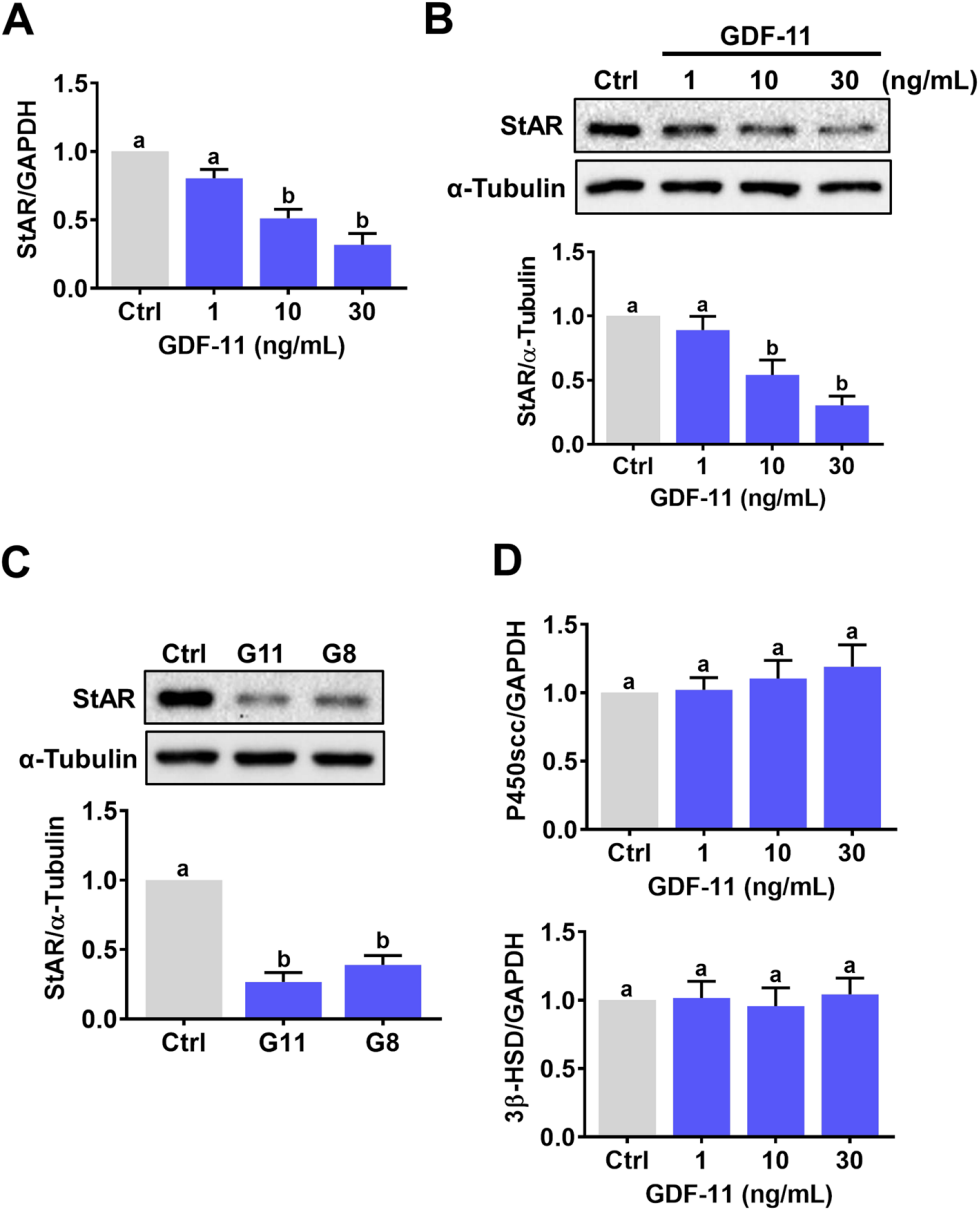
GDF-11 downregulates StAR expression in hGL cells. **A** and **B**, hGL cells were treated with 1, 10, and 30 ng/mL GDF-11 for 24 h. The mRNA (**A**) and protein (**B**) levels of StAR were determined by RT-qPCR and western blot, respectively. **C**, hGL cells were treated with 30 ng/mL GDF-11 (G11) or GDF-8 (G8) for 24 h. The protein levels of StAR were determined by western blot. **D**, hGL cells were treated with 1, 10, and 30 ng/mL GDF-11 for 24 h. The mRNA levels of P450scc and 3β-HSD were examined by RT-qPCR. The results are expressed as the mean ± SEM of at least three independent experiments. The values without a common letter are significantly different (*p* < 0.05)

In women of reproductive age, the serum GDF-11 levels can reach 40 ng/mL [24]. Therefore, to examine the effect of GDF-11 on StAR expression in hGL cells, cells were treated with 1, 10, and 30 ng/mL GDF-11 for 24 h. RT-qPCR results showed that treatment of hGL cells with 1 ng/mL did not affect the mRNA levels of StAR. However, the mRNA levels of StAR were significantly downregulated by treatments with 10 or 30 ng/mL GDF-11 (Fig. 1A). Western blot results showed the same inhibitory effect of GDF-11 on StAR protein levels (Fig. 1B). Therefore, 30 ng/mL GDF-11 was used in the subsequent experiments. Interestingly, a comparable suppressive effect was observed by treating cells with 30 ng/mL GDF-11 and GDF-8 (Fig. 1C). We also examined the effects of GDF-11 on expressions of other steroidogenesis-related enzymes. As shown in Fig. 1D, the mRNA levels of P450 side-chain cleavage enzyme (P450scc) and 3β-hydroxysteroid dehydrogenase (3β-HSD) were not affected by treatment of GDF-11.

### The suppressive effect of GDF-11 on StAR expression is mediated by ALK5 but not ALK4

**Fig. 2:**
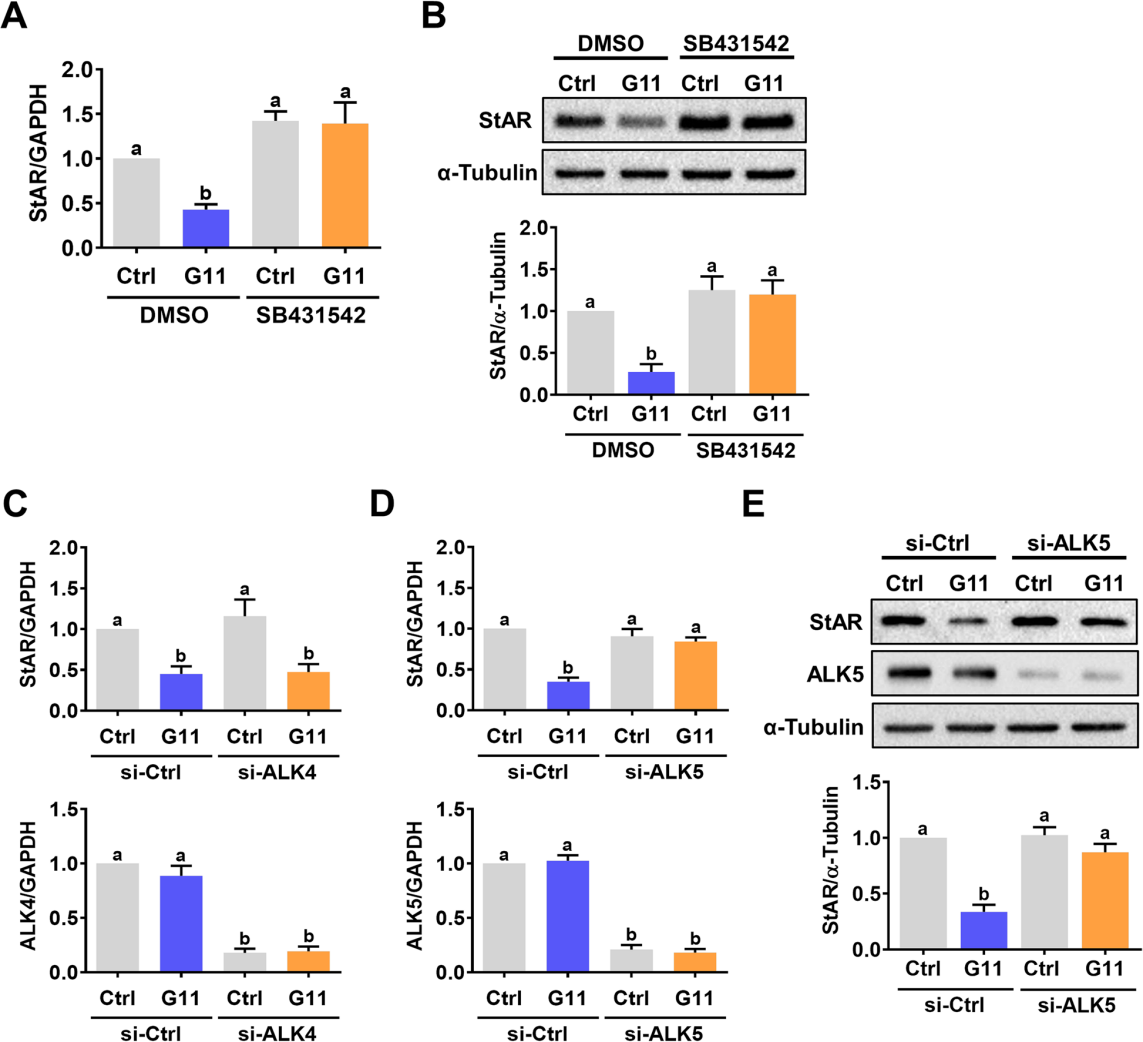
ALK5 mediates GDF-11-induced downregulation of StAR expression in hGL cells. **A** and **B**, hGL cells were pretreated with vehicle control (DMSO) or 10 µM SB431542 for 1 h, and then treated with 30 ng/mL GDF-11 (G11) for 24 h. The mRNA (**A**) and protein (**B**) levels of StAR were determined by RT-qPCR and western blot, respectively. **C** and **D**, hGL cells were transfected with 100 nM control siRNA (si-Ctrl), ALK4 siRNA (si-ALK4) (**C**), or ALK5 siRNA (si-ALK5) (**D**) for 48 h, and then treated with 30 ng/mL GDF-11 (G11) for 24 h. The mRNA levels of ALK4, ALK5, and StAR were determined by RT-qPCR. **E**, hGL cells were transfected with 100 nM control siRNA (si-Ctrl) or ALK5 siRNA (si-ALK5) for 48 h, and then treated with 30 ng/mL GDF-11 (G11) for 24 h. The protein levels of ALK5 and StAR were determined by western blot. The results are expressed as the mean ± SEM of at least three independent experiments. The values without a common letter are significantly different (*p* < 0.05)

Similar to GDF-8, TGF-β type I receptors, ALK4 and ALK5, are putative receptors for GDF-11 that mediate its biological functions [13]. Using a potent ALK4 and ALK5 inhibitor, SB431542, our results showed that inhibitions of ALK4 and ALK5 functions blocked the suppressive effect of GDF-11 on StAR mRNA and protein levels (Figs. 2A and 2B). Since SB431542 can block the functions of ALK4 and ALK5 simultaneously, to further delineate the involvement of ALK4 and ALK5 in GDF-11-inhibited StAR expression, the siRNA-mediated knockdown approach was used to block the function of ALK4 or ALK5 specifically. As shown in Fig. 2C, transfecting of hGL cells with ALK4 siRNA downregulated endogenous mRNA levels. However, the knockdown of ALK4 did not affect the suppressive effect of GDF-11 on StAR mRNA levels. In contrast, the inhibitory effects of GDF-11 on StAR mRNA and protein levels were abolished by the knockdown of ALK5 (Figs. 2D and 2E). Taken together, these results indicate that the suppressive effect of GDF-11 on StAR expression is mediated by ALK5 but not ALK4 in hGL cells.

### The suppressive effect of GDF-11 on StAR expression is mediated by SMAD3 but not SMAD2

**Fig. 3:**
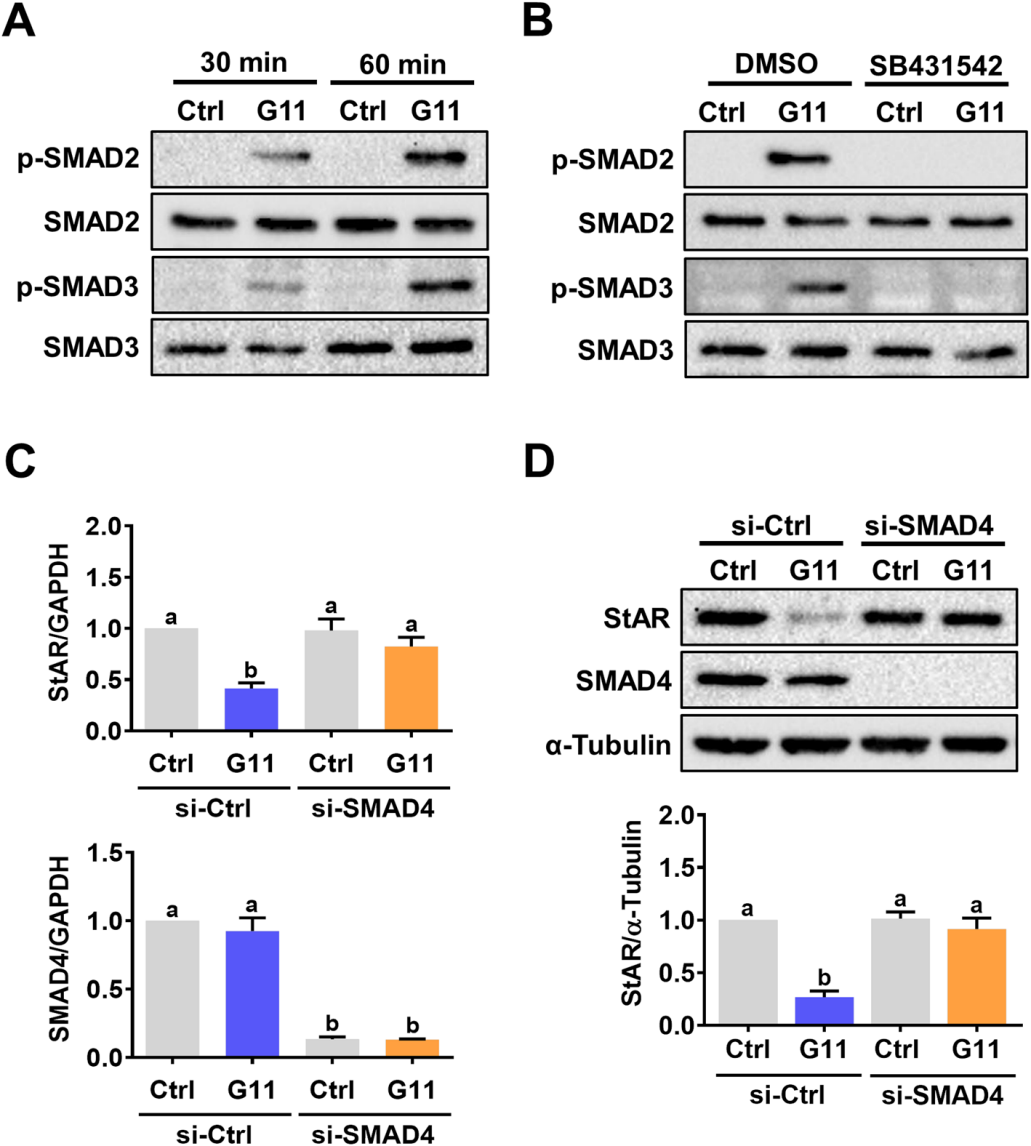
GDF-11 activates SMAD2/3 signaling pathways in hGL cells. **A**, hGL cells were treated with 30 ng/mL GDF-11 (G11) for 30 and 60 min. The levels of phosphorylated and total forms of SMAD2/3 were determined by western blot. **B**, hGL cells were pretreated with vehicle control (DMSO) or 10 µM SB431542 for 1 h, and then treated with 30 ng/mL GDF-11 (G11) for 60 min. The levels of phosphorylated and total forms of SMAD2/3 were determined by western blot. C and D, hGL cells were transfected with 100 nM control siRNA (si-Ctrl) or SMAD4 siRNA (si-SMAD4) for 48 h, and then treated with 30 ng/mL GDF-11 (G11) for 24 h. The mRNA (**C**) and protein (**D**) levels of SMAD4 and StAR were determined by RT-qPCR and western blot, respectively. The results are expressed as the mean ± SEM of at least three independent experiments. The values without a common letter are significantly different (*p* < 0.05)

**Fig. 4:**
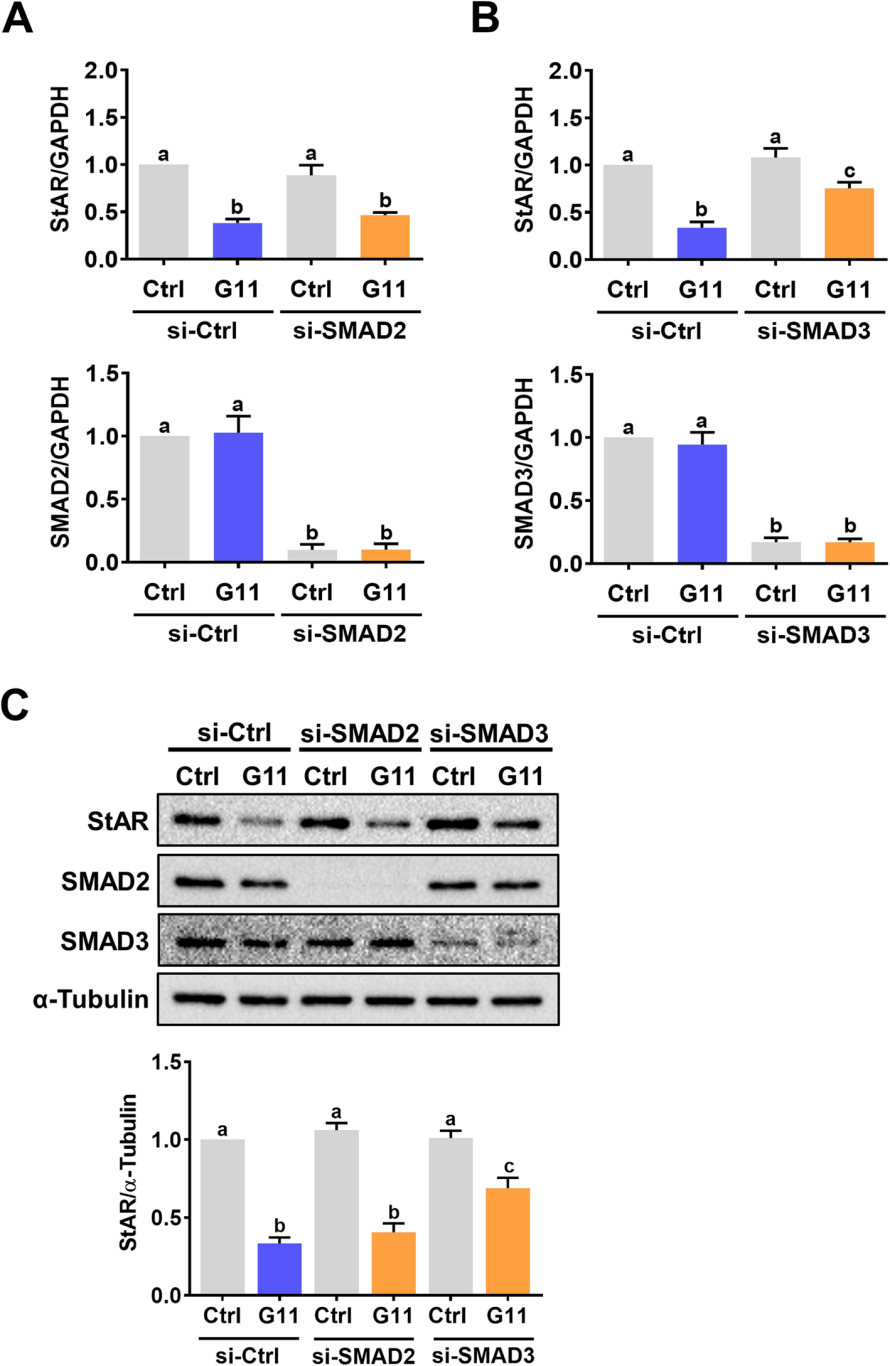
SMAD3 mediates GDF-11-induced downregulation of StAR expression in hGL cells. **A** and **B**, hGL cells were transfected with 100 nM control siRNA (si-Ctrl), SMAD2 siRNA (si-SMAD2) (**A**), or SMAD3 siRNA (si-SMAD3) (**B**) for 48 h, and then treated with 30 ng/mL GDF-11 (G11) for 24 h. The mRNA levels of SMAD2, SMAD3, and StAR were determined by RT-qPCR. **C**, hGL cells were transfected with 100 nM control siRNA (si-Ctrl), SMAD2 siRNA (si-SMAD2), or SMAD3 siRNA (si-SMAD3) for 48 h, and then treated with 30 ng/mL GDF-11 (G11) for 24 h. The protein levels of SMAD2, SMAD3, and StAR were determined by western blot. The results are expressed as the mean ± SEM of at least three independent experiments. The values without a common letter are significantly different (*p* < 0.05)

In the canonical TGF-β signaling pathway, upon ligand binding, activated ALK4/5 induces phosphorylation and activation of the receptor-regulated SMAD (R-SMAD) proteins, SMAD2 and SMAD3. Subsequently, the phosphorylated SMAD2 or SMAD3 assemble to form a complex with the common SMAD, SMAD4. This R-SMAD/SMAD4 complex translocates into the nucleus to regulate gene expression [25]. Treatment of hGL cells with GDF-11 induced phosphorylation levels of both SMAD2 and SMAD3 indicating their activations (Fig. 3A). The GDF-11-induced SMAD2 and SMAD3 activations were blocked by pretreatment with SB431542 (Fig. 3B). To examine the requirement of SMAD signaling pathways in GDF-11-induced downregulation of StAR expression, the functions of SMAD proteins were blocked by the siRNA-mediated knockdown of SMAD4. As shown in Figs. 3C and 3D, the knockdown of SMAD4 did not affect the basal levels of StAR mRNA and protein levels. However, the GDF-11-induced downregulations of StAR mRNA and protein levels were abolished by the knockdown of SMAD4. Although SMAD2 and SMAD3 are functionally interchangeable, under some conditions, SMAD2 and SMAD3 have distinct and non-overlapping roles [26]. Therefore, we next explored whether SMAD2 and SMAD3 play similar role in mediating GDF-11-downregulated StAR expression in hGL cells. To do that, SMAD2 and SMAD3 siRNA were used to knockdown endogenous expression of SMAD2 and SMAD3, respectively. As shown in Fig. 4A, the knockdown of SMAD2 did not affect the suppressive effect of GDF-11 on StAR mRNA levels. However, the GDF-11-inhibited StAR mRNA levels were attenuated by the knockdown of SMAD3 (Fig. 4B). Western blot results further confirmed the requirement of SMAD3 but not SMAD2 for the GDF-11-induced downregulation of StAR expression in hGL cells (Fig. 4C).

### GDF-11 does not activate SMAD1/5/8, ERK1/2, and AKT signaling pathways in hGL cells

**Fig. 5:**
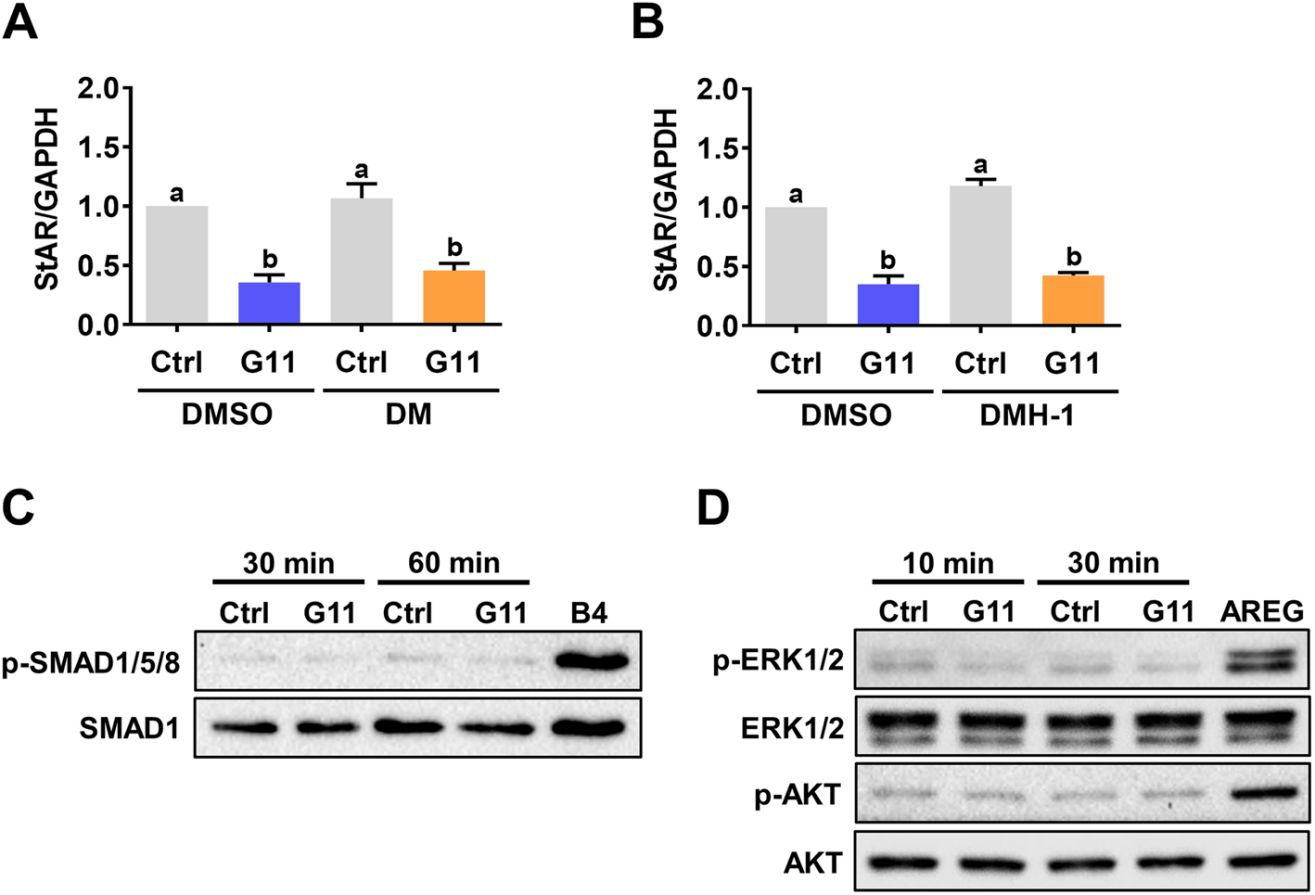
GDF-11 does not activate SMAD1/5/8, ERK1/2, and AKT signaling pathways in hGL cells. **A** and **B**, hGL cells were pretreated with vehicle control (DMSO), 1 µM dorsomorphin (DM) (**A**) or 1 µM DMH-1 for 1 h, and then treated with 30 ng/mL GDF-11 (G11) for 24 h. The mRNA levels of StAR were determined by RT-qPCR. **C**, hGL cells were treated with 30 ng/mL GDF-11 (G11) for 30 and 60 min. The levels of phosphorylated SMAD1/5/8 and the total SMAD1 were determined by western blot. Protein lysate from hGL cells treated with 10 ng/mL BMP-4 (B4) for 60 min was used as a positive control for the phosphorylation of SMAD1/5/8. **D**, hGL cells were treated with 30 ng/mL GDF-11 (G11) for 10 and 30 min. The levels of phosphorylated and total forms of ERK1/2 and AKT were determined by western blot. Protein lysate from hGL cells treated with 100 ng/mL amphiregulin (AREG) for 30 min was used as a positive control for the phosphorylation of ERK1/2 and AKT. The RT-qPCR results are expressed as the mean ± SEM of at least three independent experiments. The values without a common letter are significantly different (*p* < 0.05)

GDF-11 has been shown to activate both SMAD2/3 and SMAD1/5/8 signaling pathways in human umbilical vein endothelial cells [27]. Generally, activation of SMAD1/5/8 is mediated by the ALK1/2/3/6 [28]. Pretreatments of hGL cells with two ALK1/2/3/6 inhibitors, dorsomorphin (DM) and dorsomorphin homologue-1 (DMH-1), did not affect the suppressive effect of GDF-11 on StAR mRNA levels (Figs. 5A and 5B). Using BMP-4 as a positive control, our results showed that SMAD1/5/8 signaling pathways were not activated by the GDF-11 in hGL cells (Fig. 5C). These results together suggest that ALK1/2/3/6-SMAD1/5/8 were not involved in the GDF-11-induced downregulation of StAR expression in hGL cells. Similar to GDF-8, GDF-11 also activates some non-canonical signaling pathways such as ERK1/2 and PI3K/AKT [13]. Therefore, we also examined whether GDF-11 activates these non-canonical signaling pathways in hGL cells. As shown in Fig. 5D, using amphiregulin as a positive control, both ERK1/2 and AKT signaling pathways were not activated by GDF-11 in hGL cells.

### The levels GDF-11 in hGL cells and follicular fluid are not varied between normal and PCOS patients

**Fig. 6:**
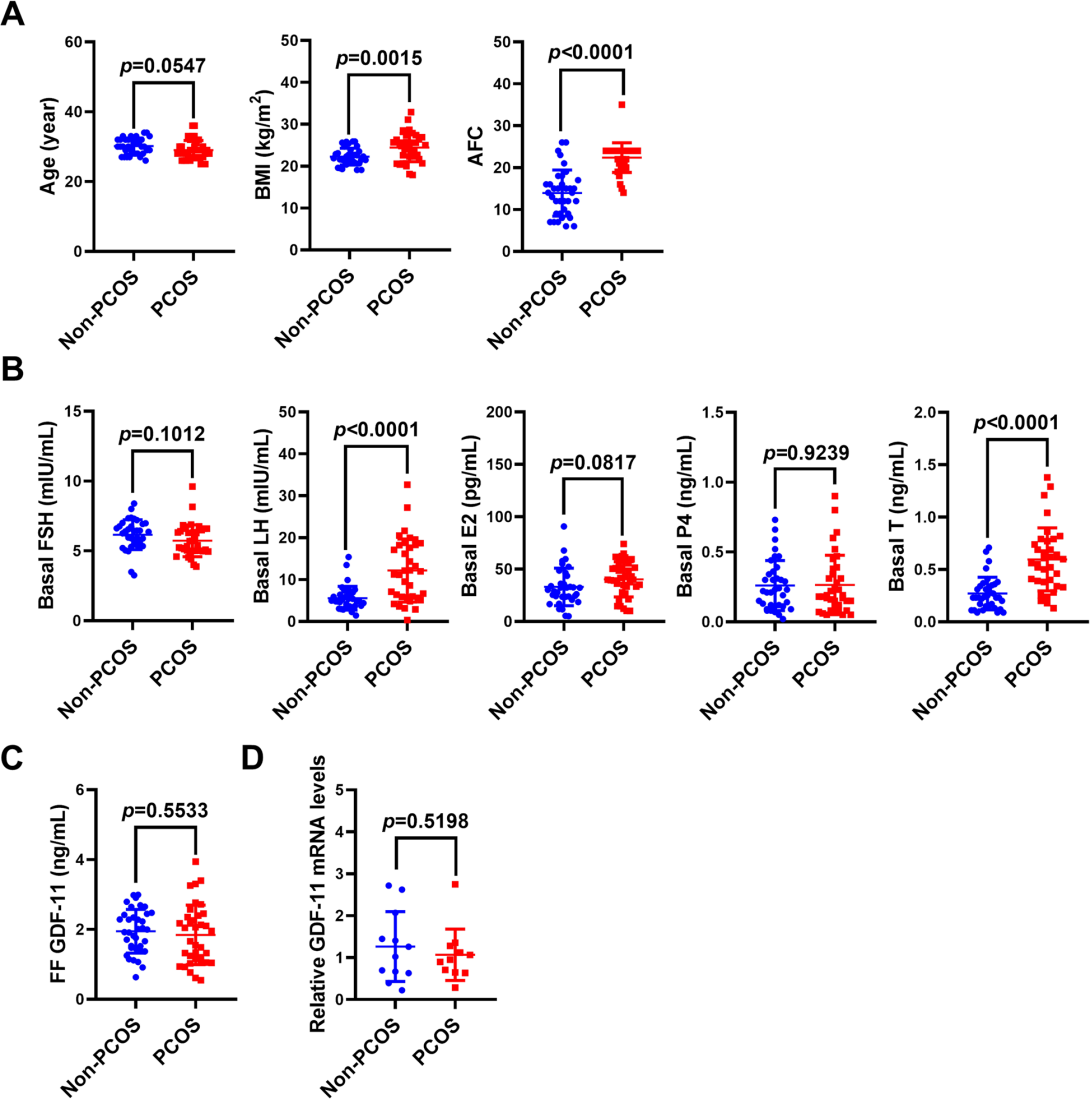
GDF-11 expression levels are not varied in hGL cells and follicular fluid between normal and PCOS patients. **A**, and **B**, Follicular fluid samples were collected from 36 non-PCOS and 36 PCOS patients of similar age. The body mass index (BMI), antral follicle count (AFC), and basal levels of hormones in serum including follicle-stimulating hormone (FSH), luteinizing hormone (LH), estradiol (E2), progesterone (P4), and testosterone (T) were measured. **C**, GDF-11 protein levels in follicular fluid samples were measured by ELISA. **D**, GDF-11 mRNA levels in hGL cells obtained from 12 non-PCOS and 12 PCOS patients were measured by RT-qPCR. The clinical results are expressed as the mean ± SD

We and another group have demonstrated that the expression levels of GDF-8 in hGL cells and follicular fluid are significantly higher in women with PCOS than in women without PCOS [16, 18, 19]. To examine whether the same is true for the GDF-11, follicular fluid samples were collected from 36 non-PCOS IVF patients and 36 IVF patients with PCOS. As expected, the body mass index (BMI) and antral follicle count (AFC) were significantly higher in PCOS patients than in control patients (Fig. 6A). In addition, the basal levels of the LH and T were increased in PCOS patients when compared to those in non-PCOS patients (Fig. 6B). ELISA results showed that the protein expression of GDF-11 was detected in the follicular fluid. However, the concentrations of GDF-11 were not significantly varied between non-PCOS patients (1.95 ± 0.626 ng/mL) and PCOS patients (1.84 ± 0.853 ng/mL) (Fig. 6C). Similarly, the mRNA expression of GDF-11 was detected in hGL cells by the RT-qPCR analysis, but the expression levels of GDF-11 were similar in both non-PCOS and PCOS patients (Fig. 6D).

## Discussion

TGF-β superfamily is composed of several subfamilies, including TGF-βs, GDFs, BMPs, activins/inhibins, anti-Mullerian hormone, and other proteins [29]. We and other groups have demonstrated that some members of the TGF-β superfamily are expressed in the human ovary and can suppress the expression of StAR in hGL cells [15, 30–35]. In the present study, we showed that similar to GDF-8, GDF-11 was expressed in hGL cells, and its protein expression was detected in human follicular fluid. In addition, our results revealed that GDF-11 suppressed StAR expression in hGL cells through the ALK5-mediated SMAD3 signaling pathway. However, in contrast to GDF-8, GDF-11 expression levels were not varied in hGL cells and follicular fluid between non-PCOS and PCOS patients.

Similar to GDF-8, GDF-11 acts mainly through ALK4/5 and downstream SMAD2/3 signaling pathways. It has been shown that the biological function of GDF-8 in myogenic cells is mediated by ALK4, while ALK5 functions as a GDF-8 receptor mainly in non-myogenic cells [36]. We have previously reported that ALK5 but not ALK4 mediates the suppressive effects of GDF-8 on StAR and pentraxin 3 expressions in hGL cells [15, 37]. In the present study, using the siRNA-mediated knockdown approach, we showed that, similar to GDF-8, the suppressive effect of GDF-11 on StAR expression was mediated by ALK5 but not ALK4. In addition to receptor level, our results also demonstrated that GDF-11-induced downregulation of StAR expression in hGL cells was mediated by SMAD3 but not SMAD2 which is the same as that observed by GDF-8 [15]. These results together with our previous findings indicate that the ALK5-mediated SMAD3 signaling pathway is required for GDF-11 and GDF-8 these two homologous proteins-induced downregulation of StAR expression in hGL cells.

Although GDF-11 is highly related to GDF-8, it is also known as BMP-11. Generally, GDF proteins signal through ALK4/5/7 and the R‑SMADs SMAD2/3, while BMP proteins signal through ALK1/2/3/6 and the R‑SMADs SMAD1/5/8 [28]. GDF-11 has been reported to activate SMAD1/5/8 signaling pathways in human umbilical vein endothelial cells [27]. However, we did not detect the same effects in hGL cells. Treatments of hGL cells with different BMP proteins activate SMAD1/5/8 signaling pathways and that is required for the downregulation of StAR expression induced by these BMP proteins [31, 35, 38]. Although we do not know the exact reasons that caused the no activation of SMAD1/5/8 signaling pathways by GDF-11 in hGL cells, this observation could not be attributed to the aberrant expression or dysfunction of ALK1/5/8. In addition to the SMAD-dependent canonical pathways, the members TGF-β superfamily also activate various intracellular SMAD-independent non-canonical pathways such as MAPK and PI3K/AKT, to reinforce, attenuate, or otherwise modulate downstream cellular responses [39]. We have shown that ERK1/2 but not PI3K/AKT signaling pathway is activated by TGF-β1 and GDF-8 in hGL cells and the activation of ERK1/2 is required for the TGF-β1 and GDF-8-induced downregulation of StAR expression [15, 33]. Treatment with GDF-11 is able to activate ERK1/2 and p38 MAPK signaling pathways in C2C12 myoblast cells [40]. Unexpectedly, neither ERK1/2 nor PI3K/AKT signaling pathway was activated in hGL cells by GDF-11 treatment. Given the high sequence similarity, it can be expected that many of the features and functions of GDF-11 and GDF-8 should be overlapped. However, a growing number of studies have described disparities in their expressions, actions, and physiological functions [13]. Therefore, further investigations into the roles of GDF-11 in the regulation of ovarian function will be of great interest.

It has been shown that circulating levels of GDF-11 are reduced in aged mice and GDF-11 can reverse age-related cardiac hypertrophy [9]. In humans, serum levels of GDF-11 in normal women (23.36 ± 2.71 ng/mL) have no significant difference from that in normal men (24.15 ± 2.40 ng/mL). In addition, serum levels of GDF-11 are not correlated with age [24]. Interestingly, another study shows that the serum levels of GDF-11 in postmenopausal Chinese women are 5.92 ± 2.52 ng/mL and increase with aging [41]. These controversial results may be due to the different methods of sample collection and measurement. In our study, there was no significant difference in age between non-PCOS and PCOS patients. Therefore, no difference of GDF-11 in the follicular fluid between non-PCOS and PCOS patients was not attributed to age. Our ELISA results showed that the levels of GDF-11 in human follicular fluid were significantly lower than those in serum. It is still unclear if GDF-11 acts predominantly as systemic hormones or if it exerts its most potent biological effects locally. Given the expression of GDF-11 in human granulosa cells and follicular fluid, it is possible that GDF-11 can act as a local factor that regulates ovarian function through autocrine and/or paracrine mechanisms. GDF-11 and GDF-8 are closely related members of the TGF-β superfamily and are often perceived to serve similar or overlapping roles. In the human ovary, GDF-8 is expressed in the oocyte, granulosa cells, theca cells, and luteal cells [16]. However, whether the same is true for the GDF-11 remains unknown. Therefore, future studies will be needed to explore the expression pattern of GDF-11 in the human ovary.

## Conclusions

In summary, the present study demonstrates the expression of GDF-11 in hGL cells and human follicular fluid and reveals a novel physiological function for GDF-11 in downregulating StAR expression in hGL cells. The suppressive effect of GDF-11 on StAR expression is mediated by ALK5 and its downstream mediator SMAD3. In contrast to GDF-8, the expression levels of GDF-11 are not varied in the ovary between non-PCOS and PCOS patients. These findings increase the understanding of the biological function of GDF-11 and provide important insights into the regulation of ovarian steroidogenesis, which may help to develop therapeutic methods for female infertility.

## Data Availability

The data that support the findings of this study are available from the corresponding author upon reasonable request.

## Abbreviations

3β-HSD: 3β-Hydroxysteroid dehydrogenase
AFC: Antral follicle count
ALK: Activin receptor-like kinase
BMI: Body mass index
BMP: Bone morphogenetic protein
DM: Dorsomorphin
DMH-1: Dorsomorphin homologue-1
DMSO: Dimethyl sulfoxide
E2: Estradiol
FSH: Follicle-stimulating hormone
GDF: Growth differentiation factor
GnRH: Gonadotropin-releasing hormone
hGL cells: Human granulosa-lutein cells
IVF: in vitro Fertilization
LH: Luteinizing hormone
P4: Progesterone
P450scc: P450 side-chain cleavage enzyme
PBS: Phosphate-buffered saline
PCOS: Polycystic ovary syndrome
StAR: Steroidogenic acute regulatory protein
T: Testosterone
TBS: Tris-buffered saline
TGF-β: Transforming growth factor-β
